# Effect of Magnetic Resonance Imaging on Breast Conservation Therapy versus Mastectomy: A Review of the Literature

**DOI:** 10.1155/2011/428653

**Published:** 2011-04-19

**Authors:** Thomas J. Painter, Peter J. DiPasco, Subhasis Misra, Eli Avisar

**Affiliations:** Division of Surgical Oncology, Dewitt Daughtry Family Department of Surgery, Sylvester Comprehensive Cancer Center, University of Miami, Miller School of Medicine, Surgical Oncology 310T, 1475 NW 12th Avenue, Suite 3550, Miami, FL 33136, USA

## Abstract

The utilization of MRI in the workup of breast cancer has played a controversial role in the surgical treatment of this disease. With the higher resolution of breast tissue afforded, additional lesions are being identified that often warrant additional procedures, subsequently affecting the decision to proceed with breast conservation therapy versus mastectomy. In this paper, a literature review is presented to help illuminate some of the benefits and pitfalls of employing MRI as a diagnostic tool in the care of breast cancer, while additionally providing insight into the management alterations this imaging modality can engender. Though further research is required in a randomized prospective form to fully answer this question, evidence for and against its use continues to mount, especially for select patient groups.

## 1. Introduction

The workup for a newly discovered breast cancer has been changing over the last decade and increasingly includes the use of preoperative magnetic resonance imaging (MRI) for staging purposes. MRI is clearly useful in selected patients: those with BRCA mutations, young women with dense breast tissue and high risk of cancer, and women with malignant axillary nodes but no evidence of a primary lesion [1]. MRI use in high-risk women (cumulative lifetime risk of 15% or more) has been shown to be more sensitive (79.5% versus 33.3%) but less specific (89.8% versus 95.0%) for detecting cancerous lesions when compared to mammography and is recommended as an adjunct for high-risk patients [2]. The American Cancer Society published an update of guidelines in 2007 which recommends routine use of MRI in patients with a 20–25% lifetime risk of breast cancer; however it does not recommend for or against screening for women with a personal history of breast cancer, carcinoma in situ, atypical hyperplasia, or extremely dense breasts on mammography [3]. Predictably, there has been a divergence in practice patterns concerning the use of MRI in the preoperative setting for these nonhigh-risk patients, and as of yet there is a paucity of substantial supporting evidence for either approach. While some have endorsed the addition of MRI to the workup of new breast cancers, others have cautioned that the technology should not be adopted until large-scale clinical trials could assess its effect on surgical management and cost [4]. Despite this controversy, many centers appear to be routinely using the technology as an adjunct to mammography and ultrasound, and research is beginning to emerge which demonstrates that it may have a deleterious effect, in that many women are undergoing unnecessary surgical procedures due to MRI findings [5–7]. Since the publication of an NIH consensus statement in 1991, breast conservation therapy (BCT) has been the preferred treatment for early-stage breast cancer [8]. Before the breast MRI era, survival has been shown to be equivalent between breast conservation surgery and mastectomy for early-stage breast cancer [9, 10]. However, providers may now be recommending additional mastectomies and even bilateral mastectomies due to findings of MRI scans [11, 12]. The aim of this paper is to examine the impact MRI has on the surgical decision of BCT versus mastectomy. 

## 2. MRI Leading to Change in Surgical Management

Because there is concern in the surgical and radiologic community that MRI may be resulting in unnecessary operations, several papers have been published in recent years which attempt to examine this interaction [13–23]. The findings of these individual studies are summarized in Table 1. In 2008, Solin et al. added to this literature by examining routine use of pre-operative MRI. Data from this study noted an increase in ipsilateral and bilateral mastectomies, more extensive lumpectomies, increased workup for patients, increased costs, and increased delays to surgery [6]. Three years before, the same author examined several prognostic elements between patients who had and had not received MRI and found no difference in 8-year rates of local recurrence, contralateral breast cancer, overall survival, cause-specific survival or freedom from distant metastases [5]. This was despite the fact that the MRI group was slightly younger and had more favorable tumor characteristics than the control group. Likewise, a group at the Mayo Clinic Rochester examined trends in their treatment of breast cancer over a ten-year period (1997–2006). They found that the general trend for mastectomy decreased over the years from 1996 to 2003 but increased again from 2003 through 2006—a trend which occurred both in the group which had received MRI and the group which had not [24]. MRI use increased drastically from the years between 2003 and 2006 as well. They noted both the surgical date and MRI as independent predictors of mastectomy in their patients. Also of note, the paper raised the issue of patient expectation, citing unpublished data from a survey conducted on 227 of their patients which revealed that 91% of patients felt the technology was reassuring and had a positive impact on their care [22].

In 2007 Bilimoria et al. published a study that suggested MRI should have a role in routine staging evaluation of newly identified breast cancers. The study found a beneficial change in surgical management in 9.7% of patients [22]. However, there was a false-positive rate of 78.0% from MRI guided biopsies, and it is possible many of these additional lesions detected by MRI may have been adequately managed by adjuvant chemotherapy and radiation. Likewise, in 2008 Grobmyer et al. suggested that routine MRI combined with MRI guided biopsies could reduce high re-excision rates. In their study, MRI use led to 25 biopsies in 79 women, 44% of which were positive for cancer, leading to a change in management for 19% of women [23]. While again the study notes a positive change in surgical management for a number of women, it is unclear whether these lesions would have been adequately treated with adjuvant chemotherapy and radiation. More concerning, the study suggests that routine MRI and frequent biopsies could become standard practice but ignores the overwhelming cost implications of this suggestion.

Other studies have not shown a significant difference between rates of mastectomy in patients who receive MRI and those that do not; however there is usually still some component of additional operations that occur in the MRI group simply due to the scan. For instance, Lim et al. contend that MRI does not lead to an increase in mastectomy; however they do admit that unnecessary surgical procedures are likely performed in patients due to benign lesions found with MRI that are indistinguishable from malignancy. In their study, surgical management changed due to use of pre-operative MRI in 84 of 535 patients (15.7%); 47 of those patients were identified as having additional malignancy while the other 37 had benign lesions [25]. The study also attempted to examine differences between patients to find those most appropriate for MRI, but analyses of age, T stage pathological diagnosis, histology, and expression of hormonal status showed no difference between groups. This study did not address the recurrence rates between groups and did not speculate on the possibility that some of the “correctly” managed patients after MRI may not have needed the additional surgery after chemotherapy or radiation. 

MRI has proven useful for detection of multifocal (lesions in the same quadrant) and multicentric (lesions in a separate quadrant) disease; however the question remains of how much of this additional detected disease is clinically relevant. A meta-analysis from 2008 found that detection of multifocal or multicentric disease ranged from 6% to 34% [26]. This is in large part a driving factor in the decision to alter surgical management; if a lesion appears on MRI to be larger or more extensive than originally thought, the surgeon frequently must alter the operative plan. In the same meta-analysis, patients in whom MRI affected surgical management ranged from 7.8% to 33.3%. The study found that 8.1% of patients were converted from BCT to mastectomy and 11.3% of patients were converted from BCT to more extensive surgery (defined as wider excision, additional excision, or mastectomy) [26]. A small percentage of these patients experienced a change in surgery due to ultimately benign lesions (false positive MRIs). The study concluded that for every two women in which MRI detected a true additional lesion, one woman would have a false positive finding in the affected breast. The authors noted that a limitation of many studies they reviewed was failure to report criteria for MRI definition of multifocal or multicentric disease, interpretation criteria for test positives, and additional information about women selected for MRI staging from the pool of all women with breast cancer. They further recommend that MRI should not be performed in centers where detection of additional lesions cannot be followed up by MRI-guided percutaneous biopsy.

## 3. Effect on Contralateral Operations

Not only does MRI lead to larger operations in the ipsilateral breast, but because it is frequently a bilateral study, contralateral operations may also be increasing as a result. Sorbero et al. published data in 2009 that demonstrated a significant correlation between MRI and contralateral prophylactic mastectomy [27]. Although their study did not evaluate causation, women who had received bilateral MRI were more than twice as likely to undergo contralateral prophylactic mastectomy. Likewise, a meta-analysis demonstrated that MRI only detected abnormalities in nearly ten percent of patients studied, less than half of which were ultimately cancerous [28]. This yielded an incremental cancer detection rate of 4.1%, PPV of 47.9%. A similar study had relatively low false positive rates for additional lesions found by MRI (6.6%); however the authors note that tissue sampling of each additional finding likely contributed to this low rate [29].

## 4. Effect on Rate of Recurrence and Reoperation

Recurrence is an undoubtedly disappointing complication of breast cancer surgery, and therefore it is essential that pre-operative imaging is able to discern the accurate tumor size. If the margins of the resected specimen are involved or are too narrow, the surgeon is obligated to reoperate—causing additional cost and emotional burden to the patient. Most studies have found that re-operation in patients who undergo pre-operative MRI is not significantly different than re-operation in patients who do not undergo pre-operative MRI [5, 7, 20, 30, 31]. It is speculated that many of the additional lesions found on MRI are adequately managed by adjuvant chemotherapy and radiation, accounting for the equivalence of recurrence in these groups [7]. One paper examined recurrence rates in women studied by MRI and those who were not and noted significantly fewer recurrences in those women who received pre-operative MRI [32]. However, the groups in the study were not matched, and those who had pre-operative MRI had less advanced lesions than the comparison group. The findings of groups who examined survival rates, recurrence rates, and margin status are summarized in Table 2.

## 5. Cost of Preoperative MRI

Another salient consideration in the use of routine pre-operative MRI is the cost of the procedure. Additionally, one must factor the attendant cost of additional biopsies and other workup when suspicious lesions are found. Very few papers address this issue directly, although some make mention of the costs associated with the technology. Moore et al. studied the issue of cost of MRI per quality adjusted life year (QALY) for women at high risk per Claus model definition and found that MRI screening did not approach cost-effectiveness even when using a threshold of $120,000/QALY [33]. Costs were estimated using Medicare and Medicaid reimbursement data. The authors additionally cited data from a similar study that examined only BRCA carriers, in which case MRI was cost effective using a threshold of $100,000/QALY.

## 6. Possible Applications

It is likely that there are specific groups that will arise in future studies who would benefit from the inclusion of MRI in the pre-operative workup of newly identified breast cancer. For instance, the use of MRI as an eligibility-screening tool for patients selected to have partial breast irradiation (PBI) is intriguing, as it could potentially prevent the undertreatment of occult malignancies. In a study by Godinez et al. from 2008 of 79 patients, it was suggested that pre-operative MRI should be performed to prevent such an unfortunate outcome [18]. These sentiments were echoed in a publication by Al-Hallaq et al. where a positive predictive value for MRI of 72% was found in identifying such occult malignancies [34]. Another recently published paper suggests a subset of patients that may have particular benefit from the addition of MRI. Mann et al. examined a patient population composed entirely of individuals with invasive lobular carcinoma (ILC) of the breast. In their retrospective cohort study, they found that patients with ILC who did not receive MRI were far more likely to undergo re-excision than the patients who had received pre-operative MRI (OR 3.64, *P* = .01) [35]. Due to the relative minority of ILC patients compared to the more common ductal carcinoma patients, it would be difficult to pursue large randomized controlled trials to further examine this phenomenon. As more researchers study MRI and its impact on patients with varied breast carcinomas, we may further elucidate patient subsets in which this technology is most appropriate.

## 7. Conclusion

MRI is a relatively new technology that has proven useful for detecting additional lesions in the pre-operative workup for breast cancer. However, while highly sensitive to additional lesions, it is less specific than mammography and results in many false positive biopsies and surgeries. It appears at the current time that the routine use of pre-operative MRI might not contribute to an increased survival and cure rates and could have a negative impact in terms of patient anxiety as well as cost to the patient and health care system. It is likely that there is a not yet delineated patient population that would greatly benefit from the use of pre-operative MRI, but as no study has been able to determine what additional criteria would help stratify those patients. It is clear from a review of the literature that randomized trials are needed to further elucidate the role MRI should play in the evaluation of women with breast cancer.

## Figures and Tables

**Table 1:** Change in Surgical Management (from breast conservation surgery) due to findings on MRI in Women with Breast Cancer.

Paper	No. of pts undergoing MRI	No. of addl lesions	% w/addl lesions	No. of addl bx	% addl bx	No. change in sx	% change in sx	True Positives	False positives
Angarita et al. [13]	71	29	40.8	7	9.9	7	9.9	Mastectomy 5/71, contralateral mastectomy 1/71, contralateral lumpectomy 1/71	NR

Lim et al. [25]	535	98	18.3	NR	NR	84	15.7	Mastectomy 21/535, wider excision 23/535, addl excision 3/535	Mastectomy 4/535, wider excision 28/535, addl excision 5/535

Scomersi et al. [29]	70	30	42.8	6	8.6	28	40.0	Mastectomy 13/70, wider excision 10/70	Mastectomy 2/70, wider excision 3/70

Pengel et al. [20]	173	19	11.0	4	2.3	19	11.0	Mastectomy 15/173, wider excision 4/173	Wider excision 1/173

Godinez et al. [18]	79	80	n/a	28	35.4	30	38.0	Mastectomy 8/79, wider excision 11/79	Mastectomy 2/79, wider excision 9/79

Grobmeyer et al. [23]	79	NR	NR	25	31.6	15	19.0	NR	NR

Bilimoria et al. [22]	155	124	80.0	41	26.5	36	23.2	Mastectomy 8/155, wider excision 5/155, contralateral 2/155	Mastectomy 2/155, wider excision 16/155, contralateral 3/155

Duerloo et al. [16]	116	48	41.4	19	16.4	25	21.6	Mastectomy 18/116, wider excision 6/116	Mastectomy NR, wider excision 1/116

Bagley et al. [14]	27	13	48.1	26	n/a	13	48.1	Mastectomy 12/155, contralateral mastectomy 1/155	NR

Liberman et al. [19]	70	36	51.4	44	62.9	20	28.6	Mastectomy 15/70, wider excision NR	Mastectomy 5/70, wider excision NR

Drew et al. [15]	178	41	23.0	3	1.7	20	11.2	NR	NR

Fischer et al. [17]	463	66	14.3	16	3.5	66	14.3	Multifocal: mastectomy 10/463, wider excision 19/463; multicentric: mastectomy 22/463; contralateral: lumpectomy 14/463, mastectomy 1/463	NR

Tan et al. [21]	83	13	15.7	NR	NR	13	15.7	Mastectomy 2/83, wider excision 3/83	Mastectomy 1/83, wider excision 7/83

*NR: not reported, bx: biopsies, sx: surgery, pts: patients.

**Table 2:** Change in Outcome in patients with MRI versus no MRI.

	Surgical outcome	Did not have MRI	Had MRI	*P*
		No. (%) with outcome	No. (%) with outcome	
Mann et al. [35]	Re-excision	25/168 (14.9)	5/99 (5.1)	.01
Turnbull et al. [7]	Re-excision/re-operation	156/807 (19.3)	153/816 (18.8)	.77
Bleicher et al. [31]	Positive margins	33/239 (13.8)	11/51 (21.6)	.20
Hwang et al. [30]	8-year recurrence	(2.5)	(1.8)	.67
Pengel et al. [20]	Positive margins	35/180 (19.4)	22/159 (13.8)	.17
Solin et al. [5]	8-year survival	471/541(87)	185/215 (86)	.51
Fischer et al. [17]	recurrence	9/133 (6.8)	1/86 (1.2)	<.001
